# Remote surgical education using synthetic models combined with an augmented reality headset^☆^

**DOI:** 10.1016/j.sopen.2022.06.004

**Published:** 2022-06-23

**Authors:** Nelson N. Stone, Michael P. Wilson, Steven H. Griffith, Jos Immerzeel, Frans Debruyne, Michael A. Gorin, Wayne Brisbane, Peter F. Orio, Laura S. Kim, Jonathan J. Stone

**Affiliations:** a Department of Urology, The Icahn School of Medicine at Mount Sinai, New York, NY; b Viomerse, Inc, Pittsford, NY; c Andros Clinics, Baarn, The Netherlands; d Urology Associates and UPMC Western Maryland, Cumberland, MD, USA; e Department of Urology, University of Pittsburgh School of Medicine, Pittsburgh, PA, USA; f Department of Urology, University of Florida Health, Gainesville, FL; g Department of Radiation Oncology, Dana-Farber/Brigham and Women's Cancer Center, Harvard Medical School, Boston, MA; h Department of Neurosurgery, University of Rochester Medical Center, Rochester, NY

## Abstract

**Objective:**

The objective was to investigate the use of an augmented reality headset to remotely train clinicians on medical devices using anatomic models.

**Design:**

Disease-specific phantoms were developed to train physicians in mpMRI-guided fusion prostate biopsy, brachytherapy, and rectal spacer insertion. Training was remotely demonstrated using 1-way virtual video conferencing format. Participants responded to an educational content survey. A heads-up display with software and augmented reality was used for remote 2-way training with the proctor and student using on their own phantoms.

**Setting:**

The virtual video meeting took place during a prostate cancer conference in 2020, while the augmented reality training occurred in 2021. The proctor and student wore a heads-up display containing a projector and webcam where the ultrasound image was displayed onto a see-through optic along with the physician's hands. The heads-up display allowed the proctor to teach by line-of-sight while the student watched and repeated the steps.

**Participants:**

Faculty with expertise with the medical devices used in these procedures provided training to urologists unfamiliar with these techniques.

**Results:**

Participants responded that the 1-way training on the phantoms was realistic and mimicked human tissue. A total of 70.9% requested more training or training on the phantoms. The remote training platform was successfully beta tested at the 2 locations in transperineal prostate biopsy and rectal spacer insertion.

**Conclusion:**

Remote training using augmented reality eliminates the need for travel. For training programs and workshops, this technology may mitigate the risk of infectious exposures, reduce training cost, and increase proctor availability, allowing training from their own institution or clinic.

This investigation qualifies for the Accreditation Council for Graduate Medical Education competency in medical knowledge.

## Highlights

• Disease and medical device specific simulation phantoms improve surgeons' skills.
• Hands-on training using phantoms can be accomplished with both instructor and student at different locations.
• A dedicated headset containing display optics and a webcam allows “line-of sight” instruction.
• Broadcasting of proctor to student and student to proctor content is optimized with specialized telecasting software which organizes the video feeds vertically.

## INTRODUCTION

Surgical education typically relies on a trainee gaining clinical experience by directly working with an instructor skilled in a specific procedure. Today, many procedures involve complex medical devices, and both resident and attending training cannot be achieved within the traditional educational framework of “See One, Do One, Teach One” [1]. This methodology should no longer be considered the standard for training because of increased procedure costs, physician and institution liability, and ultimately patient safety [1]. Several technologies have emerged to advance physician experience and education prior to introducing a new procedure into clinical practice.

Surgical instruction of residents traditionally occurs mainly around surgical cases during working hours and has been referred to as “education by random opportunity” [2]. Cadaver and animal laboratories can be used to enhance skills but lack disease-specific pathology and have limited usefulness for training on many of the newer medical devices recently introduced. For example, these models are not applicable to training on a prostate fusion biopsy platform where more realistic tissue with pathology is needed to accurately teach both image co-registration and the biopsy procedure [3]. While animal laboratories are often used for laparoscopy training, ethical considerations and the lack of disease-specific anatomy create significant barriers to their continued use and in optimizing the hands-on experience [4]. Many centers have switched to “box trainers” or synthetic bench models where components of relevant anatomy are designed to improve surgical skills. Virtual reality (VR) has also been developed for surgeons to practice and hone their skills in specific procedures or on dedicated medical devices [5].

Prior to the COVID-19 pandemic, in-person training workshops were common both for resident training and for educating clinicians on the use of new medical devices. When the pandemic hit, medical device companies were forced to stop demonstrating their technologies in person, at society meetings, and in workshops. The cancellation of the in-person American Urologic Association, European Urology Association, and American Brachytherapy Society annual meetings for 2020 and 2021 eliminated the hands-on skills workshops which were highly regarded by clinicians as an opportunity to gain experience and hone their skills on new medical devices. A detailed description of modern techniques, even with accompanying video, however, is not an optimal replacement for in-person training on models [4].

To address some of the limitations of current simulation models and the restrictions of the COVID lockdown, we created a realistic synthetic model of the prostate for performing prostate biopsy and rectal spacer insertion procedures. Additionally, we developed a remote augmented reality training platform that allows a proctor to teach these procedures to a trainee at a different location. Herein, we describe the development and testing of our synthetic prostate model as well as the remote training platform. We hypothesize that the combination of the two will facilitate resident and clinician training in surgical procedures and on new medical devices.

## MATERIALS AND METHODS

### Prostate Phantom Development and Initial Testing

The training models were developed using a customized synthetic process to create a phantom with life-like properties [[6], [7], [8], [9]]. The phantom was optimized for performing prostate biopsy, focal therapy, brachytherapy, and rectal spacer insertion. The developed model includes a prostate gland, urethra, rectum, seminal vesicles, and MRI visible lesions [10].

The prostate model was tested at a virtual prostate cancer symposium (Southwest Prostate Cancer Symposium) held on December 12, 2020. In addition to didactic lectures, virtual hands-on training was performed for transperineal biopsy using the PrecisionPoint Transperineal Access System (Perineologic, Cumberland, MD) along with the bkFusion (BK Medical ApS, Herlev, Denmark) and KOELIS Trinity (KOELIS, Inc., Princeton, NJ) biopsy platforms, and training with transrectal prostate biopsy was performed with the ARTEMIS (Eigen, Grass Valley, CA) fusion system. SpaceOAR (SpaceOAR, Boston Scientific, Boston, MA) rectal spacer insertion and Cs-131 brachytherapy (Isoray Medical, Richland, WA) seed placement were also demonstrated.

**Fig 1:**
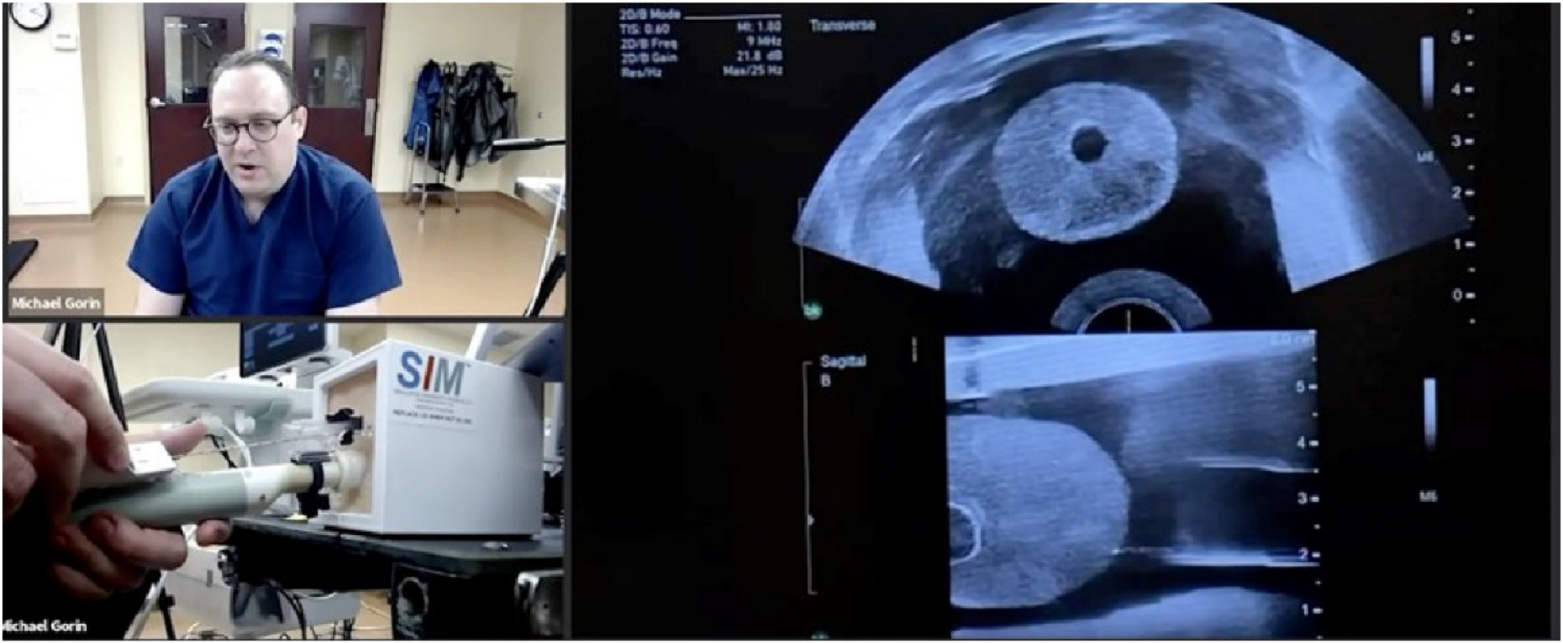
Virtual training on prostate phantom from the 2020 Southwest Prostate Cancer Symposium December 2020. Remote training was “1-way” with a proctor demonstrating a procedure to physicians watching on their laptops. A commercially available videoconferencing platform was used which presented the multiple images in a tile (horizontal) format. Proctor is teaching how to do a transperineal prostate biopsy using the PrecisionPoint transperineal access point device.

**Fig 2:**
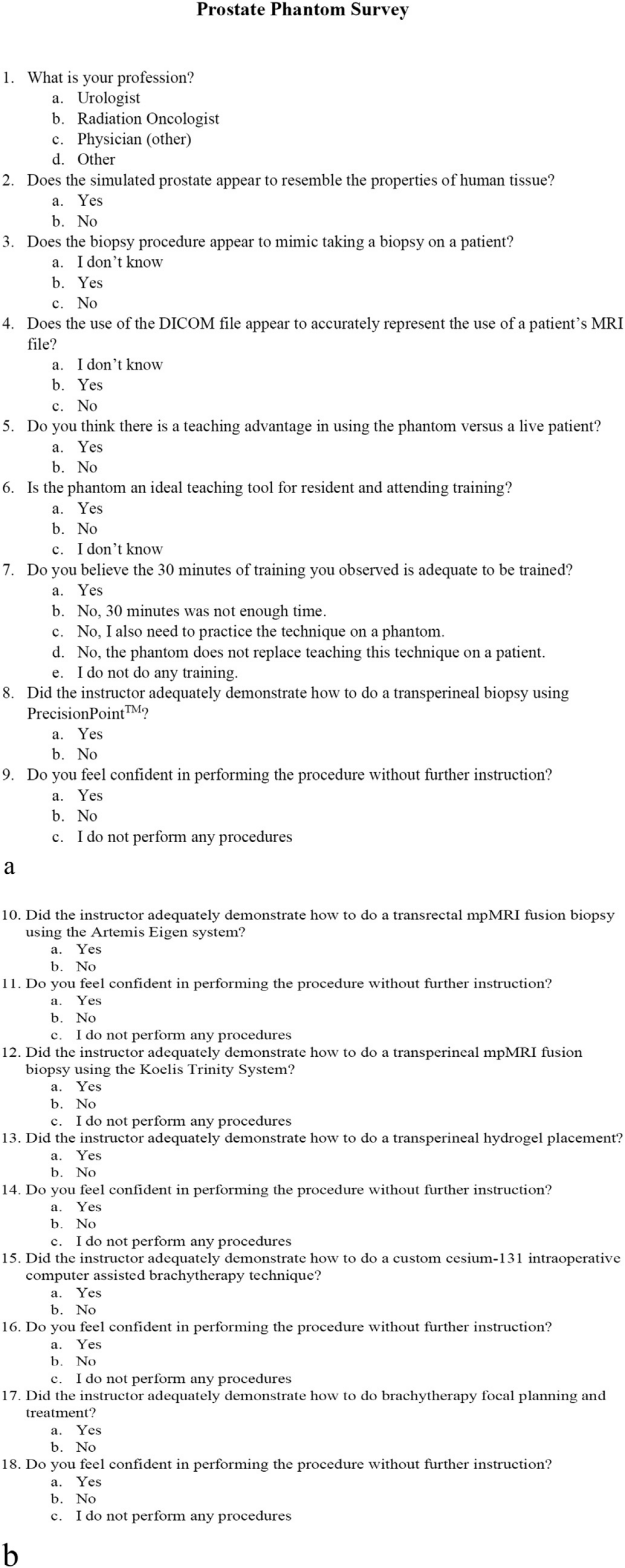
a, Prostate phantom survey (part 1). b, Prostate phantom survey (part 2).

Course faculty performed the procedures at their respective home institutions and broadcast the procedures over the internet to students via ZOOM (Zoom Video Communications, San Jose, CA). Multiple Logitech C920 webcams (Logitech International, S.A., Lausanne, Switzerland) were used to capture video of the instructors performing the procedures, and live ultrasound images were either directly displayed using an HDMI to USB device (Camlink 4K, Elgato, Munich, Germany) or filmed using a webcam. Training time was limited to 30 minutes for each procedure (Fig 1). At the completion of the course, attendees were asked to complete a survey to determine the educational value of the training (Fig 2, *a*, part 1 and *b*, part 2).

### Remote Surgical Trainer Development

**Fig 3:**
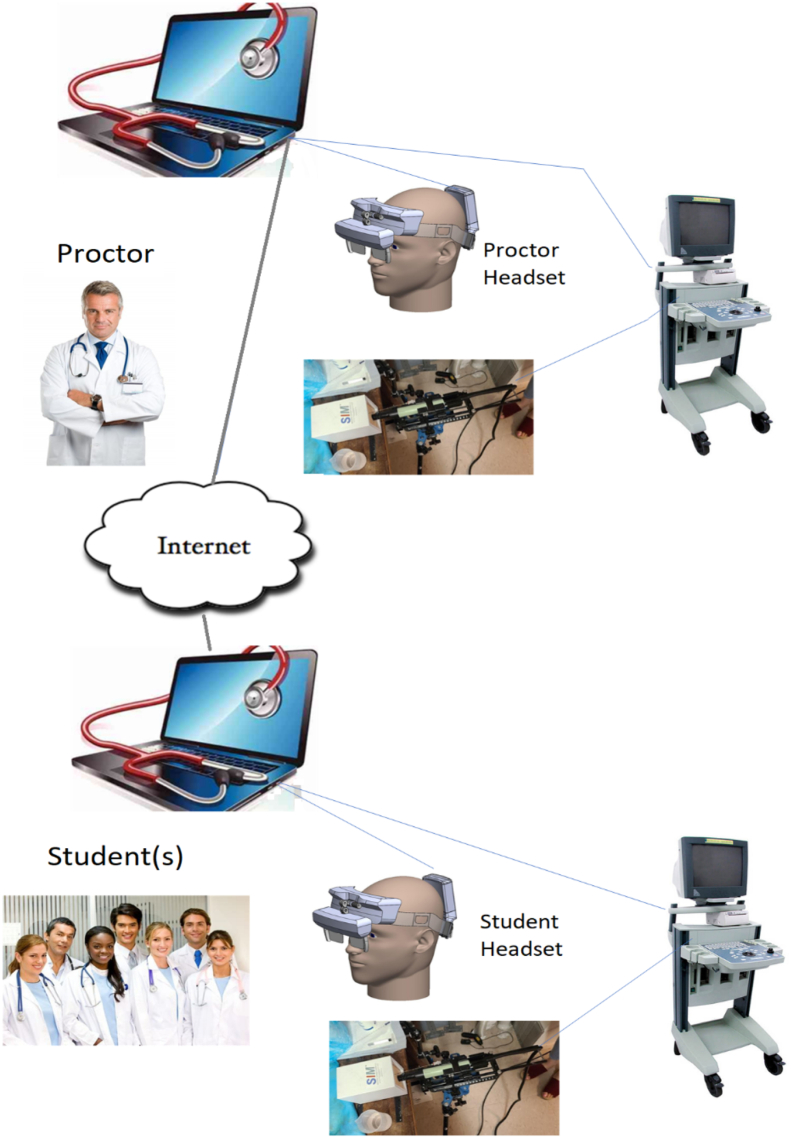
Design for the remote training platform. Video and instructional information are projected in the display optics of both the proctor and student. The proctor performs a procedure while the student observes all or portions of it followed by the student performing the same procedure on his equipment with the proctor observing and commenting on it.

To overcome the limitation of 1-way training, an augmented reality platform was developed with the intention of virtually placing the trainer in the room with the student (Fig 3). The platform includes a headset equipped with a 1920 × 1080 (full HD) resolution webcam and see-through projection optics with a 40° field of view (OE Vision optical engine and bench, Lumus, Ness Ziona, Israel). The lower portion of the optic has true see-through transparency, while the upper portion of the optic is a reflective lens that allows for projection from up to 4 video sources. The combination of the projected image and the see-through portion of the lens permits the physician to see a video image while simultaneously observing the motion of their hands.

The video output from an ultrasound unit is captured by a media hub customized for this application which is connected to a laptop. The image is in turn projected through the upper half of the optics and displayed into the physician's eye. Because of the unique design of the heads-up display, the surgeon may observe his or her hands within the operative field while simultaneously viewing the live ultrasound image. This “see-through” image may then be transmitted over the internet using proprietary video conferencing software to a student wearing a second headset. The video communication software consists of a cloud-based platform using web real-time communication (Web RTC) technology designed to use 4 separate video signals and arrange them individually over the clinician's hands in their field of view. Although no patients were investigated in this study, the video communication software is HIPPA and GPDR compliant. The proctor and student may then take turns performing the procedure, allowing the student to ask questions and the proctor to provide feedback in real time.

Following bench development of the training platform by the technical team, it was tested under real-world conditions during 2 procedures performed by physician proctors and students. The first training procedure was a transperineal prostate biopsy performed at the Andros Clinics (Baarn, The Netherlands). During this training session, the student (NNS) was “remote” to the proctor (JI); however, both were located in separate rooms within the same building. The second procedure performed was the insertion of a rectal spacer with the proctor (PFO) in Scottsdale, AZ, while the student (MPW) was located in Pittsford, NY. Each of the procedures was performed stepwise with the proctor demonstrating the first step to the student and then asking the student to repeat the next step. Following each training session, the proctor and student pairs were asked to provide feedback on their training experience.

Because the study was conducted on inanimate models outside of a university setting, no IRB approval was sought or obtained.

## RESULTS

### Initial Phantom Testing

In total, 7 faculty members and 110 students participated in the training sessions. Faculty members demonstrated the procedures from their home institutions in Maryland, Los Angeles, South Carolina, Ohio, and New York to attendees located in cities across the United States and Canada. Of these participants, 7 (100%) faculty members and 31 (28.2%) students completed the online survey. All the faculty and students believed the phantom simulated human tissue and was superior to using a live patient for training. Although 42.8% of the faculty believed that 30 minutes was adequate for training, 80.7% of respondent attendees felt more time was required, with 70.9% requesting either more training or training on the phantom themselves (Table 1).

**Table 1:** Survey results of 1-way training where proctors demonstrated procedures on prostate phantoms to physicians watching remotely. Only 7 of the 18 survey questions were analyzed

*Question*	*Faculty (*N *= 7)*			*Attendee (*N *= 31)*		
	*Yes (%)*	*No (%)*	*Other (%)*	*Yes (%)*	*No (%)*	*Other (%)*
Does the simulated prostate resemble human tissue?	7 (100)	0 (0)	0 (0)	31 (100)	0 (0)	0 (0)
Is the prostate phantom easy to set up?	7 (100)	0 (0)	0 (0)	NA⁎	NA⁎	NA⁎
Did prostate phantom biopsy mimic patient biopsy?	4 (57.2)	0 (0)	3 (42.8) Not done	23 (74.2)	0 (0)	8 (25.8) Do not know
Did the MRI file replicate a patient MRI?	6 (86)	0 (0)	1 (14) Not used	19 (61)	0 (0)	9 (29) Do not know 3(10) Missing
Is there a teaching advantage using a phantom versus a live patient?	7 (100)	0 (0)	0 (0)	26 (83.9)	5 (16.1)	0 (0)
Is the phantom an ideal teaching tool for residents and attendings?	6 (85.7)	1 (14.3)	0 (0)	24 (77.4)	2 (6.5)	5 (16.1) Missing
Do you believe that 30 min of training was adequate?	3 (42.8)	1 (14)	3 (42.8) Attendees need hands on practice	6 (19.4)	10 (32.2)	12 (38.7) Need to practice on phantom 3 (9.7) Does not replace live patient

⁎ Question only asked of faculty respondents.

### Remote Training Platform Testing

**Fig 4:**
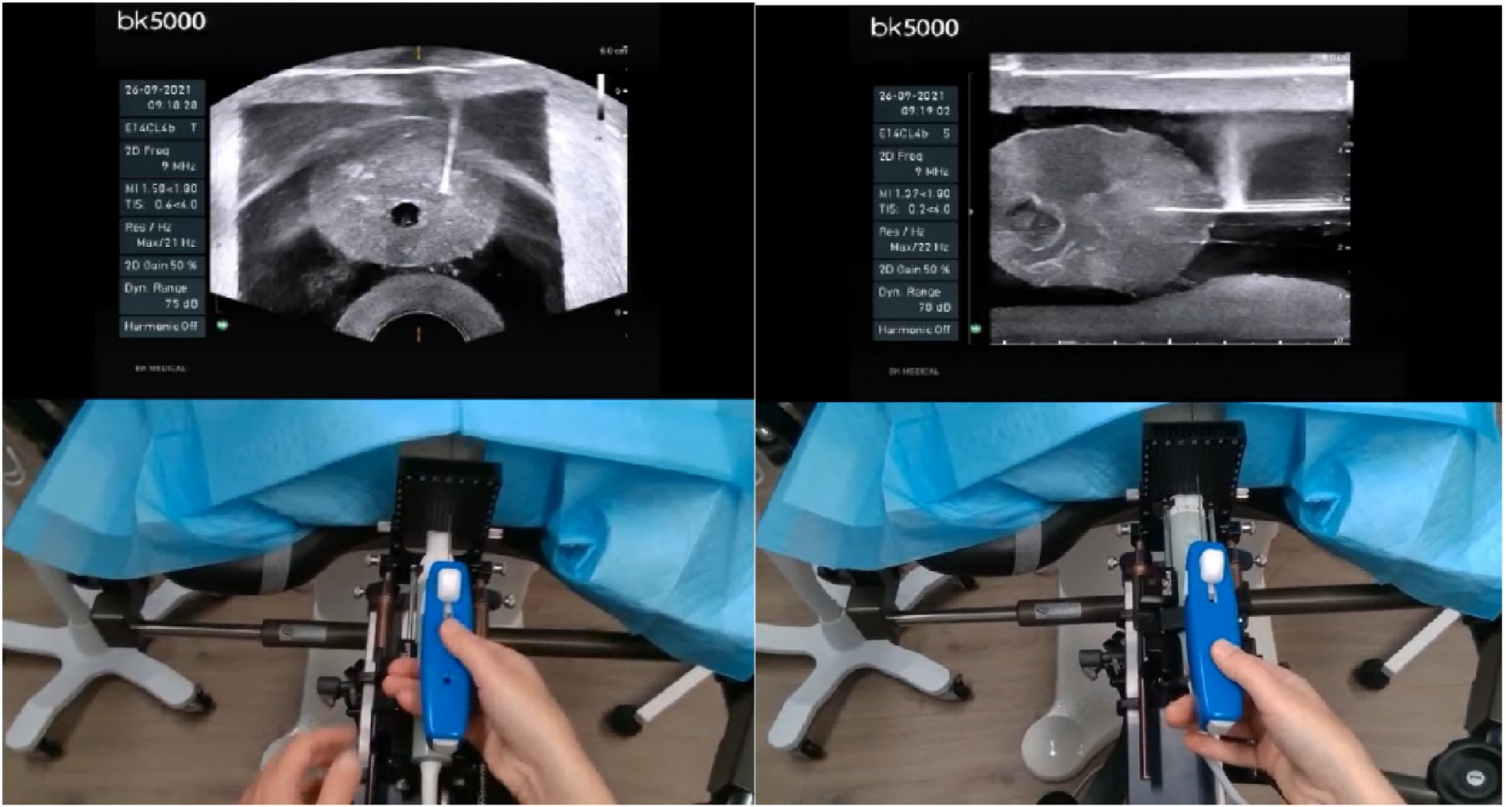
Student performs transperineal biopsy (axial and sagittal views) on prostate phantom. The student's headset displays the upper ultrasound images, while the transparent portion of the optic allows him to watch his hands and the ultrasound image simultaneously. The proctor is watching the procedure on his headset and sees these stacked images of the student's ultrasound and his hands (captured by the embedded webcam).

**Fig 5:**
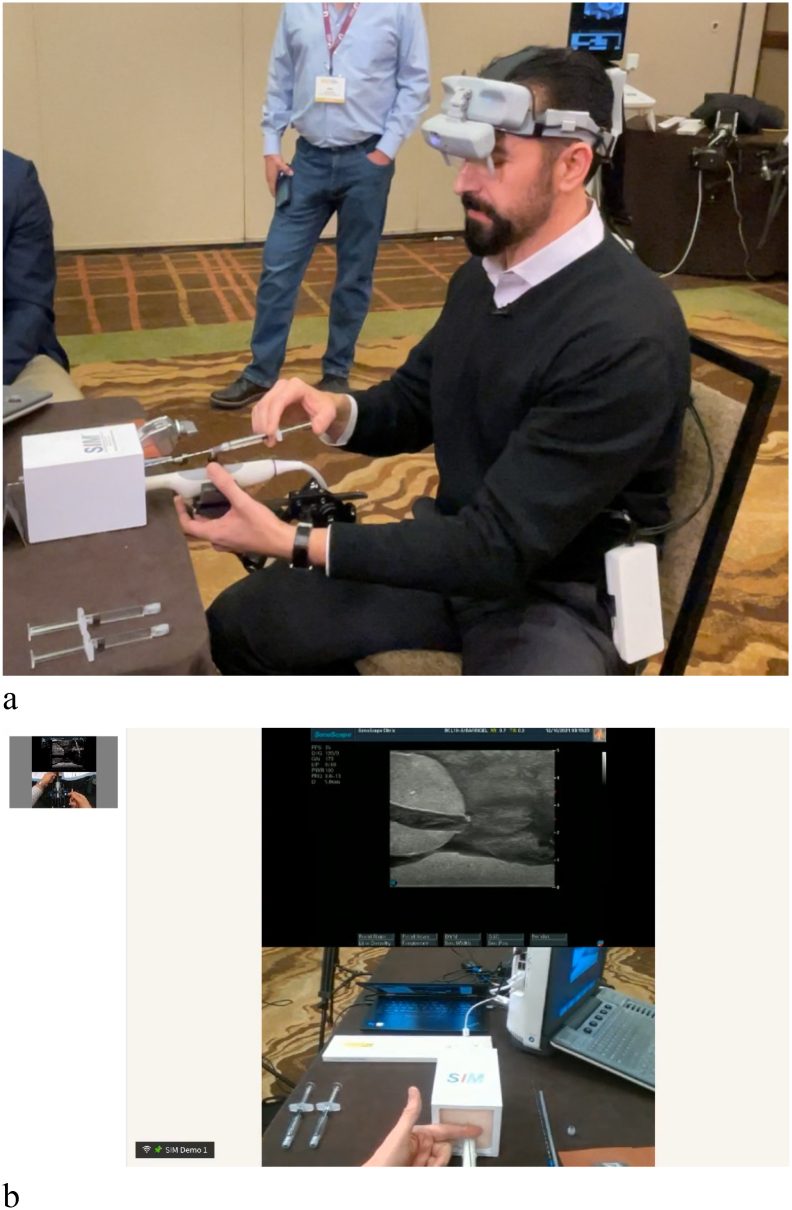
a, Proctor wears beta 3 version of headset and instructs student remotely on rectal spacer insertion. b, Proctor views ultrasound image of prostate in display optics of headset (Fig 4, *a*) while simultaneously looking at his hands during the procedure. The student can observe both the proctor's ultrasound and hands during the training in his headset.

**Fig 6:**
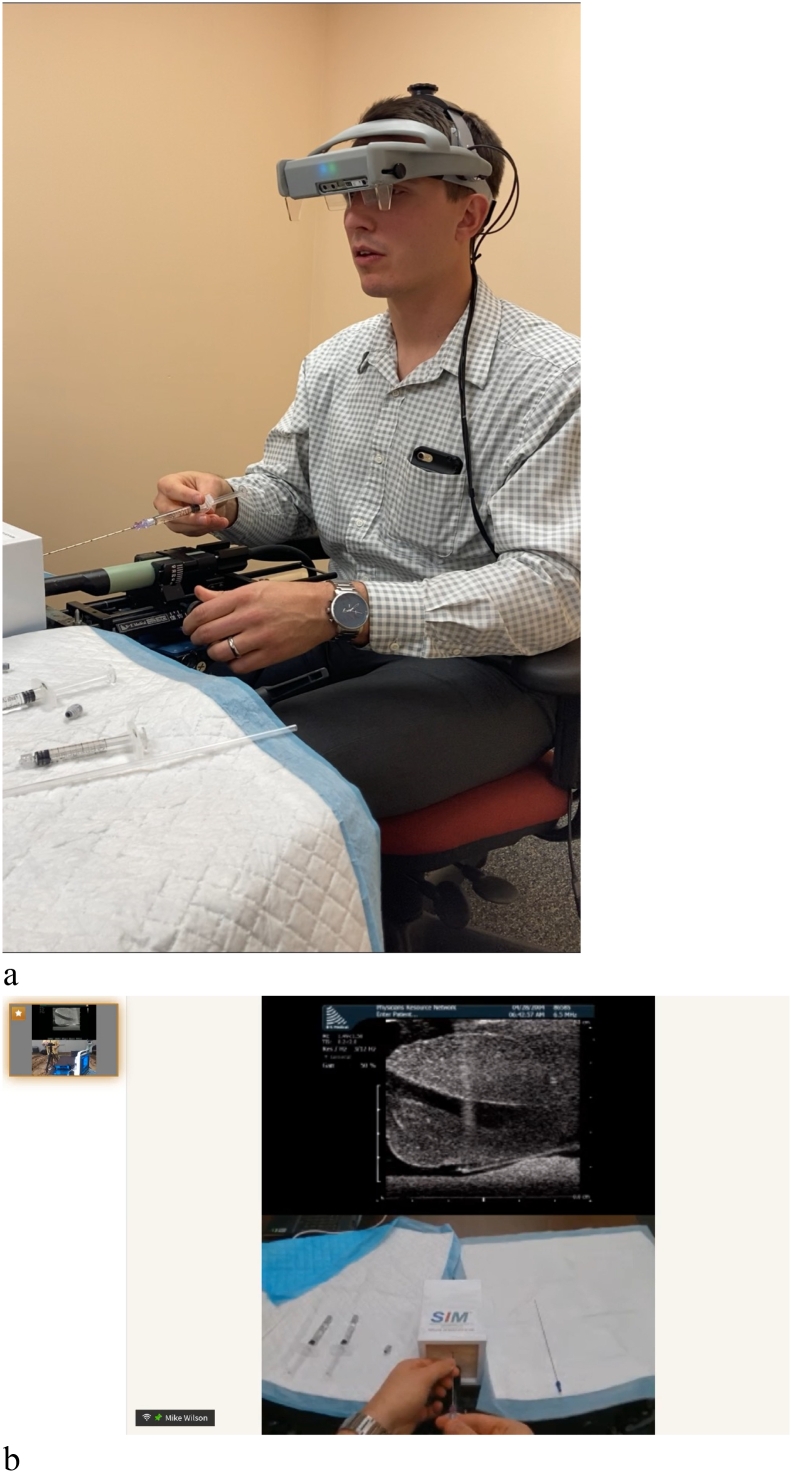
a, Student wears beta 2 version of headset while performing needle insertion prior to rectal spacer placement while proctor instructs him. b, Student views ultrasound image of prostate in display optics of headset (Fig 5) while simultaneously looking at his hands during the procedure. The proctor can observe both the student's ultrasound and hands during the training in his headset.

Testing of the remote training platform was initially performed with two beta 2 headsets at the Andros clinic with training in transperineal prostate biopsy (Fig 4). The beta 3 version of the headset was next tested where the proctor was in Scottsdale, AZ, and the student was in Pittsford, NY. The proctor trained the student how to insert a rectal spacer (Fig 5, Fig 6). In both cases, the student was able to successfully complete all steps of the procedure with real-time input of the proctor. The 4 participates (2 students and 2 proctors) felt that the images were high quality and sufficient for remote surgical training. Additionally, the participants felt that the headset was comfortable to wear and did not impede their ability to perform the procedures. The users also felt that the remote conferencing software was intuitive to use and they experienced minimal latency during the procedures. A video of the rectal spacer training can be viewed at https://youtu.be/trtTz6aAusk.

## DISCUSSION

Training of surgical residents and attendings on new medical devices has classically been done in person by proctors. In advance of their use in patients, training first on an inanimate model is an accepted means to achieve some level of competence with the procedure. Although advanced training is still done in animal or cadaveric models, many centers have moved away from these in favor of simulators that attempt to mimic live surgical conditions [11]. A variety of surgical simulators have been developed and tend to use procedure-oriented platforms [3]. For task-oriented procedures, box trainers are favored. Box and video trainers provide a relatively easy and inexpensive simulation environment for practicing minimally invasive surgery [12]. Virtual reality trainers have also emerged as a training platform; however, these training systems lack haptic feedback and for complex procedures may not provide the degree of training that can be acquired with a box trainer [13].

Three-dimensional (3D) printing technology has evolved in recent years and provides potential for developing reproducible and sophisticated physical phantoms [14,15]. More realistic phantoms can be designed which include key elements of anatomy and pathology and can be easily imaged with standard radiological modalities, allowing for their use during training procedures that require real-time image guidance. An example of this is the phantom we have described here which contains MRI visible lesions allowing students to perform co-registration of MRI and ultrasound images during a simulated prostate biopsy procedure. However, a recent review of prostate phantoms suitable for brachytherapy procedures found that only 15% of sites used commercially available models, with the most used phantom being produced by CIRS (Computerized Imaging Reference Systems, Inc., Norfolk. VA) [16]. On-site training in prostate brachytherapy using this phantom was routinely done for over 20 years by one of the authors (NNS), but the need for phantoms that maximize the potential to train on newer medical devices is clearly needed [17,18]. In addition, while on-site hands-on training proved to be highly effective, the cost and time involved make this method of teaching prohibitive. Medical device companies also struggled to find the optimal approach to training especially during the recent lock down.

The concept of remote training, with or without the use of phantoms, has been gaining traction in the medical community for several years. Remote training can be as simple as setting up several cameras in the operating room and transmitting the images to an expert who watches the procedure and provides feedback. Raborn et al looked at several remote training methodologies that could be implemented during COVID-19, which the authors termed *telesurgical mentoring* [19]. Proctors, who were fully trained surgeons, used a variety of methods including 2-way radios, video, telestration (on-screen annotations provided by the proctor), and the STAR system (System for Telementoring with Augmented Reality) [20]. Sheni et al created the VIPAR (Virtual Interactive Presence and Augmented Reality) system for remote surgical assistance [21]. This technology used 2 headsets (proctor and student) but relied on annotations to provide instruction. McCollough et al developed a platform where the student (in Africa) wore Google glasses that broadcast the operative field to a proctor [22]. Perhaps the most advanced telementoring technology to date is the STAR system developed with a grant from the military at Purdue University [23]. This system places a headset on a trainee, and the operative field is broadcast to a large monitor, allowing the proctor to overlay annotations at the surgical site.

While remote training technology has advanced, most of the training platforms have substantial limitations. These limitations provided an opportunity to develop a novel remote training platform that would be easy to use, be inexpensive, and allow for seamless interaction between proctor and student. The headset would need to be self-contained and wearable, freeing up the surgeon's hands to perform the procedure. The heads-up display we developed fulfills these needs and proved to be highly reliable in beta testing with 2 urology and radiation oncology procedures. The system allowed the proctor and student to have a shared line-of-sight view as well as the ability to visualize the other's hands with the ultrasound image in-view and overlying the phantom. An unexpected benefit of using this technology was the ability for the surgeon (proctor or trainee) to look directly at the phantom (and potentially the patient) and avoid having to look back and forth between the phantom and the monitor when performing the procedure. By having the ultrasound image superimposed on the phantom, potential procedural errors caused by averting one's gaze away from the operative site and toward the ultrasound unit was eliminated. This also allowed for improved procedural ergonomics—albeit we did not measure this in any formal manner in the presented work.

Limitations of the current study include the small sample sizes for testing of the phantom and the headset, and our lack of use of validated instruments in our assessments. Future directions include assessment of the phantom and headset for use during additional types of simulated procedures as well as testing of the headset during live cases. Outside the arena of surgical proctoring, an area of future investigation is whether use of our augmented reality headset during routine nonteaching cases can improve surgeon experience in terms of both ergonomics and visualization. Similarly, it is worth exploring surgeon interest in such technologies in general and their barriers to use.

In conclusion, we have developed the remote training platform that projects the first-person view of both the proctor and the student, allowing training to be accomplished by virtually placing the proctor “in the room” with a remotely located student. Training on inanimate models (phantoms) that more closely highlight the functionality of specific medical devices may be accomplished without the need for the trainer to travel to the student. Further, this technology should substantially decrease the cost of training while increasing its efficiency with the goal of accelerating the learning curve on new procedures prior to performing surgery on patients.

## Author Contribution

**Nelson N. Stone:** Conceptualization, Methodology, Writing – original draft, Project administration. **Michael P. Wilson:** Validation, Formal analysis, Resources, Writing – review & editing. **Steven H. Griffith:** Software, Validation, Resources, Writing – review & editing. **Jos Immerzeel:** Methodology, Validation, Investigation, Writing – review & editing. **Frans Debruyne:** Resources, Writing – review & editing, Supervision. **Michael A. Gorin:** Conceptualization, Methodology, Validation, Investigation, Writing – review & editing. **Wayne Brisbane:** Validation, Formal analysis, Investigation, Writing – review & editing. **Peter F. Orio:** Investigation, Resources, Writing – review & editing. **Laura S. Kim:** Methodology, Investigation, Resources, Visualization. **Jonathan J. Stone:** Software, Investigation, Resources, Writing – review & editing.

## Conflict of Interest

Dr Nelson N. Stone is a founder and Chief Science Officer of Viomerse, Inc. Dr Michael Wilson is a founder and Chief Executive Officer of Viomerse, Inc. Steven Griffith is a founder and Chief Technology Officer of Viomerse, Inc. Dr Michael Gorin is a consultant for Viomerse, Inc. Laura S. Kim is Vice-President for Commercial Development for Viomerse, Inc. Dr Jonathan J. Stone is founder and President of Viomerse, Inc. BK Medical (Peabody, MA) and Palette Life Sciences (Santa Barbara, CA) provided the ultrasound units and Barrigel for demonstration of the remote training platform. Viomerse, Inc. provided the prostate phantoms for the procedures. Carden Jennings Publishing (Charlottesville, VA) provided the conferencing site for the 2020 and 2021 demonstrations.

## Funding Source

There was no external funding for this investigation.

## Ethics Approval

Because the study was conducted on inanimate models outside of a university setting, no IRB approval was sought or obtained.
